# Predicting cost of care using self-reported health status data

**DOI:** 10.1186/s12913-015-1063-1

**Published:** 2015-09-23

**Authors:** Christy K. Boscardin, Ralph Gonzales, Kent L. Bradley, Maria C. Raven

**Affiliations:** Department of Medicine, Division of General Internal Medicine, University of California, San Francisco, School of Medicine, Box 3202, San Francisco, CA 94143-3202 USA; Safeway Inc., San Francisco, CA USA; Department of Emergency Medicine, University of California, San Francisco, School of Medicine, San Francisco, CA USA

**Keywords:** Cost, Health insurance, Predictive models

## Abstract

**Background:**

We examined whether self-reported employee health status data can improve the performance of administrative data-based models for predicting future high health costs, and develop a predictive model for predicting new high cost individuals.

**Methods:**

This retrospective cohort study used data from 8,917 Safeway employees self-insured by Safeway during 2008 and 2009. We created models using step-wise multivariable logistic regression starting with health services use data, then socio-demographic data, and finally adding the self-reported health status data to the model.

**Results:**

Adding self-reported health data to the baseline model that included only administrative data (health services use and demographic variables; *c*-statistic = 0.63) increased the model” predictive power (*c*-statistic = 0.70). Risk factors associated with being a new high cost individual in 2009 were: 1) had one or more ED visits in 2008 (adjusted OR: 1.87, 95 % CI: 1.52, 2.30), 2) had one or more hospitalizations in 2008 (adjusted OR: 1.95, 95 % CI: 1.38, 2.77), 3) being female (adjusted OR: 1.34, 95 % CI: 1.16, 1.55), 4) increasing age (compared with age 18-35, adjusted OR for 36-49 years: 1.28; 95 % CI: 1.03, 1.60; adjusted OR for 50-64 years: 1.92, 95 % CI: 1.55, 2.39; adjusted OR for 65+ years: 3.75, 95 % CI: 2.67, 2.23), 5) the presence of self-reported depression (adjusted OR: 1.53, 95 % CI: 1.29, 1.81), 6) chronic pain (adjusted OR: 2.22, 95 % CI: 1.81, 2.72), 7) diabetes (adjusted OR: 1.73, 95 % CI: 1.35, 2.23), 8) high blood pressure (adjusted OR: 1.42, 95 % CI: 1.21, 1.67), and 9) above average BMI (adjusted OR: 1.20, 95 % CI: 1.04, 1.38).

**Discussion:**

The comparison of the models between the full sample and the sample without theprevious high cost members indicated significant differences in the predictors. This has importantimplications for models using only the health service use (administrative data) given that the past high costis significantly correlated with future high cost and often drive the predictive models.

**Conclusions:**

Self-reported health data improved the ability of our model to identify individuals at risk for being high cost beyond what was possible with administrative data alone.

## Background

Health care accounts for an increasing percentage of the United States gross domestic product [1]. Researchers and policy makers have attempted to identify predictors of future high costs in order to develop and implement targeted interventions to reduce costs, and improve patient care. With few exceptions, risk models developed to predict future high costs have focused on Medicare, Medicaid, and VA populations [2–7]. Most studies are based on administrative data alone, while others have examined the additive value of self-reported health information [8, 9]. Previous studies have suggested that patient-reported outcomes have shown strong predictive value when predicting health care utilization and outcomes [10, 11]. However, generalization from these studies are limited by their use of non-representative sample such as VA and Medicare patients. While the ability to predict future high costs is of significant importance to local, state, and federal governments, it is also vital for large employers who self-insure and elect to provide comprehensive coverage for millions of workers.

In 2008, 89 % of workers in firms with over 5000 employees were in self-insured plans [12] and accounted for approximately 21 million workers and their dependents [13]. Many commercial insurers have expanded enrollment via participation in Health Insurance Exchanges in 2014, and will be held increasingly accountable for how health care dollars are spent [14], making cost containment and delivery of quality care of critical importance. However, the current focus on the literature on risk prediction has been on public insurance programs and thus has limited the ability of policy makers and commercial insurers to extrapolate results to privately insured patient populations that could be targeted for intervention. For example, it is suggested that factors that influence health care utilization and costs, such as poverty and social support structures, vary significantly between populations with public and private insurance. Previous studies have suggested that differences in self-reported health status between private and public insured population exhibit different expenditure patterns and health outcomes [15, 16]. To address this gap, we sought to identify predictors of future high costs within a large national commercially insured population using both more traditional administrative claims data as well as self-reported health data.

Safeway Health is Safeway’s health insurance program for non-union employees and their dependents. It is unique in its collection of self-reported health status data that provide person-level data on factors that are not normally available in administrative datasets, but may impact care-seeking behaviors. Claim-based predictive models are the most common models reported for commercially insured patients [8, 17]. Except for one recent study by Perrin et al. which focused primarily on the Medicare population which significantly limits the generalizability of the findings, the utility of self-reported data for predicting cost using commercially insured patients is unknown.

We undertook an analysis to determine predictors of future high health care expenditures for Safeway’s health plan members that included self-reported health data which included socio-demographic data such as occupation type and job satisfaction which are not typically included in the administrative data. We analyzed administrative billing data as well as biometric (e.g. BMI) and self-reported health status data from 2008 to determine the predictors of high cost in 2009. We defined high cost as being within the most costly 10 % of Safeway’s health plan members enrolled in their Choice Fund 1 and 2 plan for a given year. Our study had two aims: 1) to examine the added value of self-reported health status data in an administrative data-based model designed to identify individuals at risk for having high future health care costs and 2) to develop models that could predict which groups of individuals are at risk for being high cost in the future. In practice, high health services utilization and costs in the previous year is a very strong predictor of future high health care costs, and is information that is readily available. Therefore, we examined two types of models: one that included previously high cost members (thus identifying primarily individuals at risk of future high costs who had also been high cost in the past) and another sample that excluded members who were previously high cost, allowing us to identify predictors for being newly high cost. We hypothesized that variables present in the self-reported health status and biometric data would help to predict future expenditures in both samples.

## Methods

This was a retrospective cohort study design using insurance enrollment data from 2008 to identify patient-level factors that are associated with future high insurance costs in 2009. We used a de-identified dataset provided by Safeway. This research met criteria for exemption and approved by by the UCSF Institutional Review Board.

### Sample

The total number of employees enrolled in the Safeway health insurance program in 2009 was 18,167. Of these members, we limited our study to employees with biometric and self-reported health status data (HRQ) in 2008 since these were the key predictor variables used in our analyses. We also excluded dependents that were covered by the Safeway health insurance program through a family member (36 % of all members) since they did not complete self-reported health status data. A total of 8,917 insured employees (49 % of total number of employees) comprised our study population and were included in the analysis. The majority of the study population (78 %) resided in one of five states including: California, Oregon, Washington, Arizona and Texas. To check for possible sampling bias, we compared our study population to the population that was excluded from our analysis due to a lack of HRQ data, and examined potential differences in key demographic variables including age, gender, and occupational category (e.g. office vs. retail).

### Outcome variable

Our primary outcome was being a high utilizer of health services in 2009, defined as having total annual 2009 costs (total claims paid by Safeway’s health plan for all covered services) within the top 10 % of the study population. Individuals who are in the top 10 % of the subsequent year’s expenditure distribution have been defined as “high cost” in previous studies [18]. Sensitivity analyses from previous studies has shown that relative model performance using top 10 % as threshold seems to be consistent with the model using the top 5 % threshold [19]. We used de-identified administrative billing data from Safeway’s health plan covering the period January 1^st^ through December 31^st^ 2009 to identify the high-utilizers within the study population.

### Predictor variables

We established, *a priori,* three categories of predictor variables in the study based on the available data: 1) health services use and administrative data including number of visits to emergency department (ED) and hospitalization in 2008, and one biometric data/marker (BMI), 2) socio-demographic factors including age, sex, and the type of occupational category (facility) such as office or retail, and 3) self-reported health status from 2008 that included current and past conditions related to: back pain, chronic pain, depression, diabetes, heart problems, high blood pressure, high cholesterol and current level of job satisfaction, and fiber intake. For the self-reported health status data, responses for the condition were reported as a) never, b) previously, or c) currently. For the purposes of the analysis, we collapsed the categories previously and currently into one. The item on job satisfaction asked whether the respondent agreed or disagreed with the statement that they were satisfied with their job. For the item on fiber intake, the response options were: 1) 1-2 servings, 2) 3-4 servings, or 3) 5-6 servings.

### Analysis

To address study aim 1, which set out to examine the added value of self-reported health status data, we sequentially fitted three separate multivariable logistic regression models. We started with health services use data, then added socio-demographic data, and finally included self-reported health status data. Due to our large sample size, we used p <0.05 as the cutoff for assessing significance for each of the separate multivariable regression models. Only the statistically significant predictors from each of the three baseline models were included in the final multivariable logistic regression model. The following variables were thus excluded from the final model due to either multicollinearity or statistical insignificance: 1) among socio-demographic variables, the type of occupational category and 2) among self-reported health status variables, conditions related to: a) back pain, b) high cholesterol and c) current level of job satisfaction, and d) fiber intake.

Study aim 2 was focused on predicting which groups of individuals are at risk for being high cost in the future. To carry out this aim, we compared the final model, which included the full study sample, to a narrower sample that excluded individuals who were high cost in the previous year (top 10 % of 2008 expenditures). By excluding those who had been high cost in the past, we aimed to determine if we could isolate variables that helped predict members who would be newly high cost in 2009. First, to verify our assumptions about the necessity for subgroup analysis, we examined the strength of the interaction of each of the terms in our final model using the previous high cost status. We then developed a predictive model in this narrower sample following the same procedure outlined in aim 1. We evaluated the goodness of fit using Homer-Lemeshow chi-square test [20] and model discrimination by measuring the *c*-statistic, which is equivalent to the area-under-the-receiver operating characteristic (ROC) curve [21]. The C statistic indicates the probability that a randomly selected subject in the top 10 % utilization group will have a higher risk score than a randomly selected non-high utilizer. A value of 0.5 indicates that the model is no better than chance at making a prediction of membership in a group and a value of 1.0 indicates that the model perfectly identifies those within a group and those not. Models are typically considered reasonable when the C-statistic is higher than 0.7.

## Results

The mean paid claims amount per individual across the entire study sample for 2009 was $4,137. The high cost group (having 2009 Safeway health plan expenditures within the top 10 % of the study population) were more than twice as costly with a mean paid claims amount of $8,683. While they comprised only 10 % of the membership, they accounted for approximately 57 % of Safeway’s health plan's total expenditures for 2009. Safeway’s health plan members with HRQ data were comparable to those without HRQ data that were excluded from the study sample with respect to gender and age. However, the group with HRQ data (our study population) was more likely to work in an office setting (35 % vs. 13 %) rather than in a Safeway retail store. In our study population, about half (51 %) of high cost members in 2009 were not high cost the prior year, 2008. The characteristics of the study sample are provided in Table 1.

**Table 1:** Characteristics of Safeway Health Insurers

		2009 High Utilizer	
		No	Yes
Gender	Female	4015 (50 %)	556 (58.2 %)
Age	18-35	1849 (23.2 %)	128 (13.4 %)
	36-49	3060 (38.4 %)	294 (30.8 %)
	50-64	2826 (35.5 %)	(7.7 %)
	>65	227 (2.9 %)	77 (8.1 %)
Facility	Warehouse	363 (4.6 %)	41 (4.3 %)
	Supply	174 (2.2 %)	14 (1.5 %)
	Retail	4613 (58 %)	573 (60 %)
	Office	2809 (35.3 %)	327 (34.2 %)
Back pain	Never	4797 (60.2 %)	479 (50.2 %)
	Previously	2321 (29.2 %)	300 (31.4 %)
	Currently	844 (10.6 %)	176 (18.4 %)
Chronic Pain	Never	7457 (93.7 %)	777 (81.4 %)
	Previously	221 (2.8 %)	58 (6.1 %)
	Currently	281 (3.5 %)	119 (12.5 %)
Depression	Never	6673 (83.8 %)	682 (71.6 %)
	Previously	990 (12.4 %)	184 (19.3 %)
	Currently	296 (3.7 %)	87 (9.1 %)
Diabetes	Never	7625 (95.8 %)	853 (89.4 %)
	Previously	48 (0.6 %)	15 (1.6 %)
	Currently	285 (3.6 %)	86 (9 %)
Heart Problems	Never	7687 (96.6 %)	884 (92.7 %)
	Previously	161 (2 %)	39 (4.1 %)
	Currently	110 (1.4 %)	31 (3.2 %)
Fiber Food Intake	1-2 servings	2538 (32.4 %)	301 (32.1 %)
	3-4 servings	3938 (50.2 %)	463 (49.4 %)
	5-6 servings	1363 (17.4 %)	174 (18.6 %)
Satisfied with Job	DisAgree	587 (7.4 %)	58 (6.1 %)
	Agree	7372 (92.6 %)	897 (93.9 %)
High Cholesterol	Never	6460 (81.2 %)	668 (70 %)
	Previously	963 (12.1 %)	185 (19.4 %)
	Currently	532 (6.7 %)	101 (10.6 %)
Hospitalization	No	7788 (98 %)	908 (95 %)
	Yes	174 (2 %)	47 (5 %)
ED visit	No	7371 (93 %)	812 (85 %)
	Yes	591 (7 %)	143 (15 %)

High cost members had higher proportions of specific self-reported health conditions both during the study period and in the past, including back pain, chronic pain, depression, diabetes, heart problems, high cholesterol, high blood pressure. They also reported higher job satisfaction compared with the rest of the study population. Based on the bivariate analyses, the factors associated with being high cost (vs. low cost; all *p* < 0.001) were: 1) female gender (58 % vs. 49 %), 2) older age (49 % vs. 35 % > 50 year. old), 3) self-reported depression (23 % vs. 16 %), 4) self-reported chronic pain (12 % vs. 6 %), 5) self-reported diabetes (8 % vs. 4 %), 6) self-reported high blood pressure (29 % vs. 20 %), and 7) above average BMI (52 % vs. 42 %). The *c*-statistics for the models that excluded self-reported health variables were 0.63 (socio-demographic variables only) and 0.57 (with only prior utilization and BMI).

### Added value of self-report data

In the final adjusted model (shown in Table 2), we found that the risk factors associated with being a high cost individual, defined as having 2009 Safeway health plan expenditures within the top 10 % of the study population, were: 1) being female (adjusted OR: 1.34, 95 % CI: 1.16, 1.55), 2) increasing age (compared with age 18-35, adjusted OR for 36-49 years: 1.28; 95 % CI: 1.03, 1.60; adjusted OR for 50-64 years: 1.92, 95 % CI: 1.55, 2.39; adjusted OR for 65+ years: 3.75, 95 % CI: 2.67, 2.23),3) self-reported depression (adjusted OR: 1.53, 95 % CI: 1.29, 1.81), 4) chronic pain (adjusted OR: 2.22, 95 % CI: 1.81, 2.72), 5) diabetes (adjusted OR: 1.73, 95 % CI: 1.35, 2.23), 6) high blood pressure (adjusted OR: 1.42, 95 % CI: 1.21, 1.67), 7) above average BMI (adjusted OR: 1.20, 95 % CI: 1.04, 1.38), 8) had one or more ED visits in 2008 (adjusted OR: 1.87, 95 % CI: 1.52, 2.30), and 9) had one or more hospitalizations in 2008 (adjusted OR: 1.95, 95 % CI: 1.38, 2.77). The *c*-statistic for the final model was 0.70.

**Table 2:** Predictors Associated with high utilizers

	*Full Sample Model (n = 8917)*				*Excluding Previous High Cost Members (n = 7879)*			
	OR	Lower CI	Upper CI	*p*-value	OR	Lower CI	Upper CI	*p*-value
	Baseline				Baseline			
ED Visit	2.13	1.74	2.59	0.000	1.59	1.16	2.17	0.004
*No ED Visit*	1				1			
Hospitalization	2.10	1.51	2.93	0.000	0.76	0.27	2.09	0.589
*No Hospitalization*	1				1			
	Model 2				Model 2			
Female	1.41	1.23	1.63	0.000	1.37	1.15	1.64	0.000
*Male*	1				1			
65+	5.07	3.69	6.98	0.000	3.30	2.14	5.11	0.000
50-64	2.39	1.94	2.95	0.000	1.78	1.39	2.27	0.000
36-49	1.41	1.33	1.75	0.002	1.12	0.86	1.44	0.401
*18-35*	1				1			
ED Visit	2.09	1.71	2.55	0.000	1.62	1.18	2.22	0.003
*No ED Visit*	1				1			
Hospitalization	2.13	1.52	3.00	0.000	0.79	0.29	2.20	0.653
*No Hospitalization*	1				1			
BMI (Above Average)	1.41	1.23	1.61	0.000	1.31	1.10	1.57	0.002
*At or below average*	1				1			
	Final Model				Final Model			
Female	1.34	1.16	1.55	0.000	1.34	1.12	1.60	0.001
*Male*	1				1			
65+	3.74	2.67	5.23	0.000	2.84	1.81	4.46	0.000
50-64	1.92	1.55	2.39	0.000	1.56	1.21	2.01	0.001
36-49	1.28	1.03	1.60	0.027	1.07	0.82	1.38	0.619
*18-35*	1				1			
Chronic Pain = Yes	2.22	1.81	2.72	0.000	1.79	1.34	2.39	0.000
*Chronic Pain = No*	1				1			
Depression = Yes	1.53	1.29	1.81	0.000	1.39	1.11	1.73	0.004
*Depression = No*	1				1			
Diabetes = Yes	1.73	1.35	2.23	0.000	1.63	1.16	2.31	0.006
*Diabetes = No*	1				1			
Heart Problems = Yes	1.21	0.90	1.62	0.206	1.08	0.70	1.68	0.719
*Heart Problems = No*	1				1			
Blood Pressure = Yes	1.42	1.21	1.67	0.000	1.28	1.03	1.59	0.023
*Blood Pressure = No*	*1*				*1*			
ED visit	1.87	1.52	2.30	0.000	1.58	1.14	2.17	0.005
*No ED visit*	1				1			
Hospitalization	1.95	1.38	2.77	0.000	0.71	0.26	1.99	0.518
*No Hospitalization*	1				1			
BMI (above average)	1.20	1.04	1.38	0.014	1.18	0.98	1.41	0.075
*At or below average*	1				1			

### Predictability of the model for new high cost members

We augmented the final adjusted model to include interactions of each of its predictors with the prior high cost membership status, and this resulted in a statistically superior fit (*p* < 0.001). This suggested the potential value of a stratified analysis that examined individuals who had prior high healthcare costs separately from those who did not.

We therefore compared the predictive model that included the entire study population, including previously high cost members, to a narrower model that excluded them (thus identifying only members who were newly high cost in 2009). The predictive models that excluded previous high cost members were similar to the models that included all members but had a few notable differences. The risk factors associated with being a high utilizer in the final adjusted model for new high cost members were: 1) being female (adjusted OR: 1.34, 95 % CI: 1.12, 1.60), 2) increasing age (adjusted OR for 50-64: 1.56, 95 % CI: 1.21, 2.01; adjusted OR for ≥65: 2.84, 95 % CI: 1.81, 4.46) but only after age 49 (adjusted OR for the age group 36-49: 1.07, 95 % CI: 0.82, 1.38), 3) self-reported depression (adjusted OR: 1.39, 95 % CI: 1.11, 1.73), 4) chronic pain (adjusted OR: 1.79, 95 % CI: 1.34, 2.39), 5) diabetes (adjusted OR: 1.63, 95 % CI: 1.16, 2.31), 6) high blood pressure (adjusted OR: 1.28, 95 % CI: 1.03, 1.59), and 7) one or more ED visits in 2008 (adjusted OR: 1.58, 95 % CI: 1.14, 2.17). Finally, having above average BMI (adjusted OR: 1.18, 95 % CI: 0.98, 1.42) and one or more hospitalization in the previous year (adjusted OR: 0.71, 95 % CI: 0.26, 1.99) had wider CIs and larger *p*-values compared to the model that included previous high cost members (Model 2, Table 2). The *c*-statistic for this final model was 0.65. In summary, variables included in the narrower model were similar to those in the model of the larger population, and had the same or slightly attenuated odds ratios (e.g. gender, BMI, depression, diabetes). However, some variables were more strongly attenuated (e.g. age, ED visit), and one significant difference was found with the hospitalization variable compared to the model with full sample.

## Discussion

This is one of the first studies to examine the relative value of self-report status data in developing a predictive model for high cost using commercially insured members. In our current study, we developed a predictive model based on commercially insured patients using self-reported health status data, demographic information, and prior utilization data. We found that in addition to demographic characteristics and health services use available in billing data, the additional data collected by Safeway’s health plan related to members’ self-report of the presence of specific health conditions were of key significance for the final predictive model. Prior research indicates that patients with more than one chronic illness account for 95 % of all Medicare spending [22] and about 30 % of high-cost Medicare beneficiaries had 4 or more co-occurring chronic illnesses [14]. Despite the differences in the population, consistent with previous studies on predicting future high expenditures, chronic conditions were significantly associated with future high costs [19]. How to best accurately capture the presence of chronic conditions, however, can be complex and challenging. Certain conditions may not be coded as part of a health care encounter if they are not a primary reason for the visit. In addition, prior work shows that conditions including those we found to be key predictors of future high costs based on self-report, can be under-coded in administrative datasets [23].

Consistent with the current study Perrin et. al, also found that self-reported health status data from Medicare patients provided additional predictive power for predicting inpatient admissions and costs [8]. Previous studies have indicated that self-reported health status provides important data that can aid in prediction of future costs. Some have provided evidence that a single item assessing general self-rated health (GRSH) has robust predictive power regarding future expenditures for selected samples [24, 25]. Yet to date, little research has examined the use of member reported health and biometric data in commercially insured populations to predict future costs. In a study with nationally representative data from the Medical Expenditure Panel Survey, DeSalvo et. al, [24] was able to obtain a *c*-statistic of 0.85 for predicting the upper 10 % of total expenditures with a model including diagnostic cost group (DCG) system. DCG models use patient demographic information such as age, sex, and medical diagnoses obtained from insurance claims databases to determine risk scores. One reason for the lower c-statistic in our study may have been the lack of diagnoses data in the model. Adding an in-depth examination of cost-drivers using procedure logs, diagnostic codes, and frequency of hospitalizations could improve the prediction model and provide an effective intervention protocols to target specific patient characteristics. But it is also likely that these procedures overlap substantially with previous health care utilization/costs. Our study shows that moderate predictive power can be achieved by including 9 factors recorded using a combination of self-reported, biometric, and past health services utilization data. This simpler algorithm provides an alternative to other cost predictive models.

The cost of collecting self-reported health data must be weighed against the cost of using administrative or biometric data for risk prediction: in some cases, the ease of obtaining readily-available administrative data may weigh against collecting additional self-reported data from health plan members. However, the utility of self-reported data reaches beyond predictive power alone, and can help provide information that will facilitate intervention development and adoption at the individual patient level. There are likely multiple uses of self-reported health data beyond predictive modeling, which can help payers better understand the population they are covering. Accurate prediction of high utilization is desirable because failure to allocate resources properly can generate biased treatment effects and can lead to over- or under-payment for certain types of patients. The logistical challenges of collecting self-reported data may be unfeasible in practice, however, some have argued that the collection costs has been overstated and application a recently developed limited-sample benchmark method can help to ease the burden on the resources required for accurate prediction.

Prior health services utilization pattern such as ED visits and hospital admissions were also predictive of high cost in the subsequent year, consistent with past research focused on Medicaid beneficiaries.[3, 7] Given that predictive models based on claims data are often driven by past high cost members with specific condition and utilization patterns (i.e. ED visits and hospitalization), the analysis excluding previous high cost members provides additional information on the key predictors. In the narrower predictive model, having one or more ED visits in the prior year was a significant risk factor for being high cost in the future. The lack of significance of hospitalization in the narrower model could be attributed to the exclusion of the past high utilizers which can be also considered proxy for high cost hospitalization. For example, 68 % of those in the high utilization group in 2008 were hospitalized. Additionally, the significance and the predicted value of the self-report health status data in predicting new high cost members provide a strong rationale for collecting self-report data to optimize patient-centered outcomes and can allow payers to target new potential high cost members that are often missed using only the prior utilization data. These results clearly have programmatic implications for identifying high-risk patients for appropriate case or enhanced management programs. From a clinical perspective, reducing revisits to ED by tracking frequent ED patients, contacting the primary care provider while the patient is in the ED and focusing on better communication, proactive transitions and collaboration between ED and primary care providers can all help to reduce over utilization of services in the future.

In our study, the comparison of the models between the full sample and the sample without the previous high cost members indicated significant differences in the predictors. This has important implications for models using only the health service use (administrative data) given that the past high cost is significantly correlated with future high cost and often drive the predictive models.

Our study has limitations. While we used a national cross-section of Safeway Health members, because our model is based on data from a single commercial plan, the findings may not be generalizable across other employer plans with different patient populations. However, our results are in keeping with previous research in this area. Our study sample was limited to Safeway’s health plan enrolled employees with HRQ data. However, the comparability of the study sample with the non-study sample provide minimize potential bias in our study. Given that we used self-reported data, the potential for errors in omission and response bias exist. Previous studies in the UK have indicated potential for response bias using self-reported data due to significant false negative reporting on chronic condition such as hypertension and lack of sensitivity to multiple measures of health due to over reliance on a single indicator related to the level of vitality (energy) [26, 27]. However, previous studies have also shown that using self-report comorbidity indices, although different from HRQ, performed similarly to predictive models using administrative medical records data providing additional case for reliability of the data [28, 29].

## Conclusion

Despite these limitations, our study has clear implications for enhanced management and health care delivery outcomes. Given the predictive power of the self-reported health status, patient demographics, and past health services utilization patterns, the relative low cost of administration and applicability of the method across variety of care delivery settings may provide an alternative to more complicated DCG models. Whenever possible, commercial plans should attempt to include these data in research and reporting efforts as they will be important for identifying high-risk, high cost patients and developing interventions to improve care. This model may be a viable alternative for systems where other data are not available or too costly to collect.
